# Tolerability and performance of BIP endotracheal tubes with noble metal alloy coating – a randomized clinical evaluation study

**DOI:** 10.1186/s12871-015-0156-z

**Published:** 2015-12-01

**Authors:** Gunilla Björling, Dorota Johansson, Linda Bergström, Shah Jalal, Ivar Kohn, Claes Frostell, Sigridur Kalman

**Affiliations:** 1 The Swedish Red Cross University College, P.O. Box 55676, SE-10215 Stockholm, Sweden; 2 Karolinska Institutet, Department of Clinical Sciences, Division of Anaesthesia and Intensive Care, Danderyd Hospital, SE-18288 Stockholm, Sweden; 3 Bactiguard AB, Stockholm, Sweden; 4 P.O Box 15, SE-146 21 Tullinge, Sweden; 5 Karolinska Institutet, Division of Clinical Microbiology, Department of Laboratory Medicine, SE-14186 Stockholm, Sweden; 6 Department Anesthesia and Intensive Care, Karolinska University Hospital Huddinge, SE-14186 Stockholm, Sweden; 7 CLINTEC, Karolinska Institutet, SE-14186 Stockholm, Sweden

**Keywords:** Endotracheal tube, Colonization, bacterial, Complication, Clinical method, Anaesthesia, Airway, Noble metal alloy coating

## Abstract

**Background:**

Hospital acquired infections worsen the outcome of patients treated in intensive care units and are costly. Coatings with silver or metal alloys may reduce or alter the formation of biofilm on invasive medical devices. An endotracheal tube (ETT) is used to connect the patient to a ventilator and coated tubes have been tested in relation to bacterial colonization and respiratory infection. In the present study, we aimed to evaluate and compare a coated and uncoated ETT for patient symptoms and local tracheal tolerability during short term clinical use. Degree of bacterial colonization was also described.

**Methods:**

A silver-palladium-gold alloy coating (‘Bactiguard®’Infection Protection, BIP) has been extensively used on urinary tract catheters and lately also on central venous catheters. We performed a randomised, single-blinded, controlled, first in man, post Conformité Européenne (EC) certification and CE marking study, focused on Bactiguard® coated ETTs (BIP ETT). Thirty patients at a tertiary university hospital scheduled for upper abdominal elective surgery with an expected duration of anaesthesia of at least 3 h were randomised; BIP ETT (*n* = 20) or standard ETT (*n* = 10). The tolerability was assessed with a modified version of Quality of Life Head and Neck Module, QLQ-H&N35 and by inspection of the tracheal mucosa with a fibre-optic bronchoscope before intubation and at extubation. Adverse Events (AE) and bacterial adherence were also studied. Statistical evaluations were carried out with the Fisher’s Exact Test, the Clopper-Pearson method, as well as a Proportional Odds Model.

**Results:**

Differences between groups were identified in 2 of 8 patient related symptoms with regard to tolerability by QLQ-H&N35 (cough, *p* = 0.022 and dry mouth, *p* = 0.014 in the treatment group.). No mucosal damage was identified with bronchoscopy. A low level of bacterial colonization with normal flora, equal between groups, was seen after short-term of intubation (median 5 h). No serious Adverse Events related to the use of an ETT were observed. The results should be treated with caution due to statistical confounders, a small study size and large inter-individual variability in bacterial adhesion.

**Conclusions:**

The new device BIP ETT is well tolerated and has good clinical performance during short-term intubation. Studies with larger sample sizes and longer intubation periods (>24 h) in the ICU-setting are needed and can now be planned in order to identify possible differences in clinical outcomes.

**Trial registration:**

Registered in ClinicalTrials.gov, Registration number: NCT01682486, Date of Registration: August, 30, 2012

## Background

Hospital acquired infections (HAI) are a global problem and among the four major causes of death in the Western world. Generally, 10 % of hospitalised patients in the Western world acquire HAI [1–4]. Medical devices are rapidly colonized with bacteria during the formation of biofilm, which in turn can lead to health care-related infections [5–8]. But as the usage of medical technical equipment is necessary within the health care system, it is a particularly important area to study and optimize [9, 10].

To reduce the biofilm formation on endotracheal tubes, coatings with pure silver or silver compounds have been evaluated. The efficacy and safety of endotracheal tubes (ETTs) coated with pure silver or silver compounds have been reported in three randomised controlled clinical [11–13]. No safety issues were reported. The studies reported significant reduction of biofilm formation [12, 13] and as well as a delay of VAP. The noble metal alloy coating has been extensively used on urinary catheters and central venous lines [14–16]. This is a first in man study on an endotracheal tube with the Bactiguard® noble metal coating (BIP ETT).

The primary objective was to determine the tolerability of the medical device BIP ETT compared to standard uncoated ETTs in a small selected population of patients undergoing elective surgery. The secondary objective was to assess the overall safety and performance related to the medical device. The hypothesis was that the coated tube (BIP ETT) would be similar to the standard endotracheal tube with regard to patient-experienced symptoms and that the tracheal mucosa would be similarly affected by contact with the BIP ETT as by contact with a standard endotracheal tub. The primary end-point was patient-reported symptoms in the throat and mouth, as well as visual appearances of the upper tracheal mucosa the day after tracheal intubation.

Though bacterial colonization is of major interest when it comes to medical devices inserted in the human body, this study is focused on the tracheal safety and tolerability when exposed to the new coating. Bacterial colonization after a few hours of endotracheal tube usage is described here and can be considered as a baseline in future studies with coated endotracheal tubes used for longer clinical periods (days).

## Methods

### Study design

This was a single-centre, randomised, single-blinded, and controlled, first in man, post Conformity Euro-permit (EC) certification and CE marking study. Patients undergoing elective surgery of the upper gastrointestinal tract with an expected duration of at least 3 h were asked to participate. The study was conducted at a Karolinska University Hospital in Sweden and approved by the Ethical Review Board in Stockholm (Dnr 20121205–31, protocol number 1842-2026-CDOC). Written informed consent from each patient was obtained before entry into the study. All patients signed a written informed consent before participating in the study. The study is outlined in clinicaltrials.gov database with the identifier: NCT01682486.

### Investigational device BIP ETT

BIP ETT is an endotracheal tube designed for insertion through the mouth or nose. The, in- and outside surfaces are coated with the Bactiguard® coating (Sweden) which consists of a noble metal alloy of silver, palladium and gold, which reduces bacterial adhesion in-vitro. The latter phenomenon is a contributing factor to microbial colonisation and biofilm formation, which in turn increases the risk for VAP. The BIP ETT is made from medical grade polyvinyl chloride (PVC). It is a sterile single use 100 % latex free, two-lumen tube (one lumen connected to a high volume low pressure cuff with a standard connector), has a Magill curve and rounded tip with a Murphy eye.

### Control device

Standard uncoated ETTs made of PVC, normally used for intubation at the investigators site (Mallinckrodt™ ETT) with standard labelling, were used as reference devices. They were sterile single use 100 % latex free, two-lumen tubes (one lumen connected to a high volume low pressure cuff with a standard connector), had a Magill curve and rounded tip with a Murphy eye.

### Study population

Inclusion criteria were adults ≥18 years of age, requiring endotracheal intubation ≥3 h needing an endotracheal tube sized 7 or 8 mm. Exclusion criteria were cognitive dysfunction (i.e., inability to understand and fill out the study questionnaires), transmissible blood disease, multi-resistant bacterial colonization, current or continuous treatment with immune modulating therapies, e.g., systemic (or inhalation) use of cortisone or non-steroid anti-inflammatory drugs (NSAID), on-going respiratory infection, as well as deficient poor skills in writing and or speaking Swedish.

### Screening and randomization

The list of scheduled major surgery eligible patients were identified and scanned and thereafter screened by reading the patients’ journal. Eligible patients were later approached for information and consent. In total, 30 patients were screened and 30 patients were enrolled in the study. The enrolled patients were randomised to airway management during anaesthesia and surgery with either the BIP ETT (*n* = 20) or standard ETT (*n* = 10). The randomisation was designed to distribute the patients in a ratio of 2:1 in favour of the BIP ETT group. One patient in the BIP ETT group was prematurely withdrawn from the study due to vomiting during induction of anaesthesia. The patient then needed a rapid sequence induction and therefore, pre-intubation bronchoscopy could not be carried out. Therefore, the patient was withdrawn from the study.

### Endpoints

#### Primary endpoints

The difference between the treatments (BIP ETT versus standard ETT) for each of the 8 questions in the modified European Organization for Research and Treatment of Cancer (EORTC) Quality of Life Questionnaire for Head & Neck with 35 questions (QLQ H&N35) [18], on the day after tracheal intubation.

The difference between treatments (BIP ETT versus standard ETT) in photo assessment (blinded assessment), and direct assessment (unblinded assessment) of the surface tracheal lining, before intubation and after extubation.

#### Secondary endpoints

Safety assessed based on adverse events (AE) and serious AEs (SAEs) and on any problems occurring during the post-operative course (Yes/No).

Performance assessed by bacterial colonization of BIP ETT versus standard ETT, microbiological testing of bacteria from a defined area of the ETT surface *ex vivo* (quantification and typing).

### Data collection

Demographics, smoking habits, medical history of significance, and concomitant medication were assessed prior to surgery. The study comprised of four visits; a telephone contact by a study nurse to inform the patient about the study and send the written patient information to the patient up to 28 days prior to screening (Visit 1); a screening visit 1 to 14 days prior to surgery when also the informed consent form was signed and demographic data, medical history gathered and physical examination was performed (Visit 2); the actual day of surgery/intubation (Visit 3), and a follow-up on the day after surgery (Visit 4).

The study intervention was carried out at Visit 3. As the patient arrived in the operating theatre, local routines were followed. No lidocaine or lubricating jellies were used in conjunction with the ETT. Prior to intubation of the ETT, the endotracheal cuff was inflated as a test. The intubation was carried out after a bronchoscopy where the ETT was pulled over the fiberscope, and the cuff pressure was kept below 20 cmH_2_O if possible. Endotracheal cuff pressures were recorded using an intra-cuff monitor, and the pressure was recorded during the anaesthesia. After induction of anaesthesia, the number of intubation attempts and the need for other intubation devices were recorded, as well as any injuries to lip, tongue or teeth. The ETT was not to be repositioned unless necessary. During extubation, a bronchoscope was again placed in the trachea through the ETT. The ETT was then removed and the trachea inspected. The time for intubation and extubation, as well as the type of fibre-optic bronchoscope were noted. The medical techniques and aseptic procedures used were according to currently accepted practice.

#### Assessments of tolerability

To assess the tolerability of the BIP ETT, a modified version of the head and neck module of the questionnaire developed for assessing quality of life was used (Quality of Life Head and Neck Module, QLQ-H&N35) [18]. The questionnaire was originally developed by the European Organization for Research and Treatment of Cancer (EORTC) [19]. In the present study, eight of the questions, which assessed symptoms that can occur after intubation, were used. Permission to use the adjusted version was obtained from EORTC. The selected questions were the following (the original question number in the EORTC QLQ H&N35 within parenthesis):

- Have you had pain in your mouth? (31)
- Have you had pain in your jaw? (32)
- Have you had soreness in your mouth? (33)
- Have you had a painful throat? (34)
- Have you had problems swallowing liquids? (35)
- Have you had a dry mouth? (41)
- Have you coughed? (45)
- Have you been hoarse? (46)

Each patient indicated the extent to which he/she had experienced these symptoms or problems as “Not at all”, “A little”, “Quite a bit” or “Very much”. The questionnaire was completed by the patients at 2 time points in time; prior to surgery (Visit 3, to cover the past 24 h) and in the morning of the first post-operative day (Visit 4, to cover the period post-surgery). The baseline was established before surgery in order to be able to distinguish between the intensity of symptoms in relation to the tube alone and not the patient’s condition prior to surgery.

In order to detect any changes in the tracheal mucosa correlated with the ETT, visual inspections were carried out by an anaesthesiologist/surgeon both before intubation and after extubation using a flexible fibre-optic bronchoscopy. The assessments were formulated using the following 4-graded scale:

I. No signs of irritation/inflammation of the mucosa in the trachea
II. Weak signs of irritation/inflammation of the mucosa in the trachea
III. Clear signs of irritation/inflammation of the mucosa in the trachea
IV. Extensive signs of irritation or extreme discoloration/inflammation of the mucosa in the trachea

#### Assessments of performance and safety

Safety and Performance were assessed after surgery (Visit 3 and Visit 4) by noting Adverse Events, occurrence of complications during the post-operative course and any ETT handling problems experienced by the staff. It was the duty and right of the investigator to interrupt the patient’s participation if the patient’s health or well-being was threatened by continuation in the study. A patient could be discontinued if e.g., wrong ETT was placed by mistake. Further, concomitant medications could require a patient’s discontinuation. However, each case of withdrawal was to be discussed individually with the Sponsor prior to any decision of discontinuation, unless the decision was based on safety reasons. The pre-defined reasons for patient withdrawal were as follows:

1. Serious life threatening AE, related or unrelated to the treatment in test (i.e., the ETT). This could be events such as critical bleeding, myocardial infarct, urticaria, angioedema chock and asthma bronchiale.
2. Patient’s own request
3. Investigator’s opinion that it is in the patient’s best interest to discontinue. The reason, time and date of discontinuation had to be recorded in the eCRF.

#### Exploratory assessments of microbial adhesion to endotracheal tubes

Immediately after extubation, the distal piece of the ETT was cut with a sterile pair of scissors precisely above the cuff into a sterile tube containing PBS (Phosphate Buffered Saline) covering the tube to keep microorganisms alive. The container was immediately transported to the Department of Clinical Microbiology, to assess the quantity and type of pathogen and/or non-pathogen microbes. The antibiotic resistance of pathogen microbes (if those were found) was also to be determined.

At arrival in the lab, the tube was vortexed vigorously in order to remove mucus from the ETT piece. The ETT piece was then moved to a new tube containing 30 mL of PBS, washed by vortexing and if needed repeatedly washed until all visual mucus was removed. Thereafter it was put in another tube containing 10 mL PBS and 0.05 % Tween-20. In order to remove the adhered microorganisms from the surface it was vortexed for 1 min at maximum speed. The PBS-Tween solution with the removed microorganisms was diluted and plated out according to standard procedures for clinical airway sampling at the Dept. of Clinical Microbiology, and evaluated by viable count and microbiological typing the following day [20–22].

### Statistical analysis

In this exploratory study, 30 patients (20 patients in the BIP ETT group and 10 in the uncoated, standard ETT group) were judged to be needed in order to reveal statistically significant differences in the primary analyses. With an estimated drop-out rate of 20 %, the expected number of evaluable patients was 24. At the end, 29 patients were evaluated (19 in the BIP ETT group and 10 in the standard ETT group).

For the 8 questions used from the EORTC QLQ H&N35, the difference between the treatment groups on the day after surgery were tested with Fisher’s Exact Test. Confidence intervals (95 %) of the proportion of patients for each treatment were calculated using the Clopper-Pearson method. The questions after surgery were analysed with a Proportional Odds Model, specifying a multinomial distribution and cumulative logit link. The model included the answer of the respective questions before surgery as a covariate, and tested the difference between the treatments using the likelihood ratio statistic. The proportional odds assumption was tested with the Score test. If more than 4 of the 8 score tests rejected the null hypothesis of proportional odds, then an extended Mantel-Haenzel analysis controlling for the EORTC QLQ H&N35 answer before surgery were to be applied for each of the questions.

Differences between the treatment groups of assessed changes in the tracheal mucosa, on the 4-graded scale, were analysed using Fisher’s Exact Test. Confidence intervals (95 %) of the proportion of patients for each treatment were calculated using the Clopper-Pearson method.

Direct assessment after extubation was also to be analysed using a Proportional Odds Model, including the evaluation/judgement prior to intubation as a covariate. However, since all patients in the standard ETT treatment group were in the same category at extubation, the Proportional Odds Model analysis was not applicable.

Adverse events (AEs) and adverse device related events (ADEs) were coded using MedDRA version 15.1. No statistical tests were carried out but an overall summary of the number and proportion of patients and the number of events in each AE category (AE, SAE (Serious AE), ADE, SADE (Serious ADE) was made. However, since only one AE was reported, no tabulation of AEs/ADEs was carried out. Problems during the post-operative course are presented with descriptive statistics by treatment group (number and proportion of patients). Problems related to the ETT handling were presented in descriptive statistics by treatment group as the number and proportion of patients in each category (Yes/No).

## Results

All patients, who had completed the study (*n* = 29), were included in the analysis set; *n* = 19 in BIP ETT treatment group and *n* = 10 in the standard ETT treatment group.

### Demographics

The demographics, weight and height, are given in Table 1.

**Table 1:** Demographics and baseline characteristics

		Coated ETT	Standard ETT	Total	*p*-value
		*n* = 19	*n* = 10	*n* = 29	
Age	Mean (SD)	67.3 (7.3)	60.8 (13.7)	65.1 (10.2)	0.19
Sex	Female	13 (68 %)	5 (50 %)	18 (62 %)	0.43
	Male	6 (32 %)	5 (50 %)	11 (38 %)	
Weight (kg)	Mean (SD)	72.1 (16.2)	72.0 (9.7)	72.0 (14.1)	0.98
Height (cm)	Mean (SD)	166.7 (8.5)	172.6 (8.5)	168.7 (8.8)	0.09
Smoking Status	Never smoked	13 (68 %)	6 (60 %)	19 (66 %)	0.84
	Stopped	4 (21 %)	3 (30 %)	7 (24 %)	
	Yes, currently	2 (10 %)	1 (10 %)	3 (10 %)	

*Max* Maximum, *Min* Minimum, *Q1* First quartile, *Q3* Third quartile, *SD* Standard deviation

Percentages are based on the number of subjects within each treatment group

No deviations were found for any of the patients in either treatment group regarding physical examination or vital signs prior to surgery. In total, 4 patients in the study had concurrent diseases or relevant medical history, see Table 2.

**Table 2:** Medical/surgical history and concurrent diseases

	Coated ETT	Standard ETT	Total	*p*-value
	*n* = 19	*n* = 10	*n* = 29	
System Organ Class/Preferred Term	n (%)	n (%)	n (%)	
Immune system disorders	2(10)	1(10)	3(10)	1
Allergy to metals	1 (5)	1(10)	2 (7)	
Drug hypersensitivity	1 (5)	0	1 (3)	
Seasonal allergy	0	1(10)	1(3)	
Respiratory, thoracic and mediastinal disorders	1(5)	0	1(3)	1
Cough	1(5)	0	1(3)	
Surgical and medical procedures	0	1(10)	1(3)	
Coronary artery bypass	0	1(10)	1(3)	0.34

*n* = Number of patients reporting the term

### Tolerability evaluation

#### Modified EORTC QLQ H&N35

*Before surgery*, none of the patients from either study group reported any pain in their mouth, jaw or throat, nor did anyone report any swallowing problems. One patient in the BIP ETT group reported soreness in the mouth, 5 %. With regards to cough one case in the BIP ETT-group (5 %) and in two cases in the control ETT group (20 %) were reported a substantial number of patients experienced dry mouth before surgery i.e., 63 % in BIP ETT group and 50 % in the standard ETT group. Thus, both groups reported similar symptoms from the throat area before the surgery.

The difference between responses from the BIP ETT group and standard ETT group on the day *after surgery* was tested using Fisher’s Exact Test. The outcome after surgery is reported in the Table 3.

**Table 3:** Modified EORTC QLQ H&N35. Differences between treatment groups after surgery using the Fisher’s Exact Test

		Coated ETT		Standard ETT		
		*n* = 19		*n* = 10		
Parameter	Analysis value	n (%)	95 % CI^a^	n (%)	95 % CI^a^	*p*-value^b^
Cough	Not at all	9 (47)	(24; 71)	7 (70)	(35; 93)	0.6294
	A little	9 (47)	(24; 71)	3 (30)	(7; 65)	
	Quite a bit	0	(0;18)	0	(0; 31)	
	Very much	1 (5)	(0; 26)	0	(0; 31)	
Dry mouth	Not at all	0	(0; 18)	1 (10)	(0; 44)	0.043
	A little	0	(0; 18)	2 (20)	(2; 56)	
	Quite a bit	6 (32)	(13; 57)	4 (40)	(12; 74)	
	Very much	13 (69)	(43; 88)	3 (30)	(7; 65)	
Hoarse	Not at all	5 (26)	(9; 51)	2 (20)	(2; 56)	0.883
	A little	9 (47)	(24; 71)	4 (40)	(12; 74)	
	Quite a bit	3 (16)	(3; 40)	3 (30)	(7; 65)	
	Very much	2 (10)	(1; 33)	1 (10)	(0; 44)	
Pain in jaw	Not at all	19 (100)	(82; 100)	10 (100)	(69; 100)	
	A little	0	(0; 18)	0	(0; 31)	
	Quite a bit	0	(0; 18)	0	(0; 31)	
	Very much	0	(0; 18)	0	(0; 31)	
Pain in mouth	Not at all	19 (100)	(82; 100)	9 (90)	(56; 100)	0.345
	A little	0	(0; 18)	1 (10)	(0; 44)	
	Quite a bit	0	(0; 18)	0	(0;31)	
	Very much	0	(0; 18)	0	(0; 31)	
Painful throat	Not at all	13 (68)	(43; 88)	9 (90)	(56; 00)	0.590
	A little	5 (26)	(9; 51)	1 (10)	(0.3; 44)	
	Quite a bit	1 (5)	(0.1; 26)	0	(0; 31)	
	Very much	0	(0; 18)	0	(0; 31)	
Problems swallowing liquids	Not at all	19 (100)	(82; 100)	8 (89)	(52; 100)	0.333
	A little	0	(0; 19)	0	(0; 34)	
	Quite a bit	0	(0; 19)	0	(0; 34)	
	Very much	0	(0; 19)	1 (11)	(0.3; 48)	
Soreness in mouth	Not at all	19 (100)	(82; 100)	10 (100)	(69; 100)	
	A little	0	(0; 18)	0	(0; 31)	
	Quite a bit	0	(0; 18)	0	(0; 31)	
	Very much	0	(0; 18)	0	(0; 31)	

^a^Confidence Interval ^b^Fisher’s Exact Test

Fisher’s Exact Test was not performed for pain in jaw and soreness in mouth since all patients were in one category

A statistically significant difference was found for dry mouth (*p*-value: 0.0434), suggesting more problems with dry mouth in the BIP ETT treatment group. As mentioned, a large proportion (63 %) of these patients reported symptoms before surgery. No statistically significant difference was found for the other symptoms/problems. Fisher’s Exact Test was not carried out for pain in the jaw and soreness in mouth since all patients were in the same category.

An analysis with Proportional Odds Models controlled for value/category prior to surgery for cough and painful throat symptoms. But due to non-convergence, not for the value prior to surgery concerning pain in the jaw and dry mouth. There was a statistically significant difference between the treatment groups for cough *p* = 0.022 and for dry mouth symptoms *p* = 0.014, suggesting somewhat more problems with these aspects in the BIP ETT treatment group compared to the standard ETT group, see Table 4. Since more patients in the BIP ETT group had symptoms prior to surgery, regarding the dry mouth symptom this difference should be treated with caution.

**Table 4:** Modified EORTC QLQ H&N35 Difference between treatments day after surgery, Proportional Odds Model

Parameter	Odds Ratio^a^	95 % CI^b^	*p*-value
Cough	0.082	0.003, 0.726	0.022
Dry mouth	0.14	0.02, 0.67	0.014
Hoarse	1.56	0.38, 6.62	0.535
Painful throat	0.24	0.01, 1.71	0.165

^a^Odds ratio between coated ETT and standard ETT (ratio of the odds of being in the lower categories). The model controls for baseline for the parameters Cough and Painful throat but due to non-convergence, baseline was not included in the Dry mouth and Hoarseness models. The Proportional Odds Model was only applied to the symptoms/problems when not all patients within a treatment group were in the same category

^b^Likelihood ratio based confidence interval

There were no signs of irritation or inflammation of the upper airway surface tracheal lining at intubation or extubation, in the standard ETT group. In the BIP ETT group, weak signs of irritation/inflammation were seen at intubation for one patient (5.6 %). At the time of extubation, 4 patients had weak signs of irritation/inflammation. In one of the cases, however, − the evaluation prior to surgery was missing and in a second case, the patient had showed irritation already prior to surgery. There was no statistically significant difference between the BIP ETT and standard ETT treatment group (*p*-value: 0.268), see Table 5.

**Table 5:** Upper airway unblinded judgement, Fisher’s Exact Test

		Coated ETT		Standard ETT		
		*n* = 19		*n* = 10		
Parameter	Analysis value	n (%)	95 % CI^a^	n (%)	95 % CI^a^	*p*-value^b^
Intubation	I	17 (94)	(73, 100)	10 (100)	(69 % 100)	.
	II	1 (6)	(0, 27)	0	(0, 31)	.
	III	0	(0, 19)	0	(0, 31)	.
	IV	0	(0, 19)	0	(0, 31)	.
Extubation	I	15 (79)	(54, 94)	10 (100)	(69, 100)	.
	II	4 (21)	(6, 46)	0	(0, 31)	.
	III	0	(0, 18)	0	(0, 31)	.
	IV	0	(0 %, 18 %)	0	(0 %, 31 %)	0.2680

^a^Confidence intervals calculated using Clopper-Pearson method. ^b^Fisher’s Exact Test. Only provided for extubation

I - No signs of irritation/inflammation of the mucosa in the trachea; II - Weak signs of irritation/inflammation of the mucosa in the trachea; III - Clear signs of irritation/inflammation of the mucosa in the trachea; IV - Extensive signs of irritation or extreme discoloration/inflammation of the mucosa in the trachea

### Safety and performance evaluation

One AE was reported for one patient in the BIP ETT group during the course of the study.

A shallow wound was seen in the trachea prior to extubation, probably caused either by the mechanical impact of the fiberscope and/or the BIP ETT according to the anaesthesiologist. It was not considered a result of a difficult airway hence the patient was not excluded from the analysis set. No signs of inflammation were noted. The start date of the event was reported as the day of surgery (Visit 3), and the event was on-going at the time of the patient’s study completion. The event was considered mild in severity and non-serious. No medication or treatment was given due to the AE.

ETT handling problems were reported for 1 patient (5 %) in the BIP ETT group: there were some difficulties when pulling the ETT over the fibreoptic bronchoscope. This was however not considered serious.

### Exploratory assessment of microbial adhesion to endotracheal tubes

**Fig. 1:**
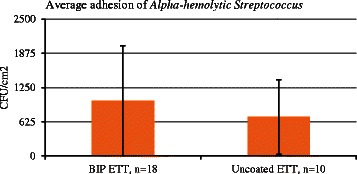
*In vivo* colonization of *Streptococcus* presented as CFU/cm^2^. Standard deviation is displayed

**Fig. 2:**
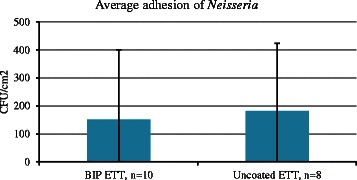
*In vivo* colonization of *Neisseria* presented as CFU/cm^2^. Standard deviation is displayed

The microbiological analysis of the ETTs after extubation showed *Enterococci* species, *Haemophilus parainfluenzae*, *Neisseria species*, *Staphylococci* and *Streptococci species*, see Table 6. These species are all recognized as normal flora in the airways, i.e., no pathogenic species were found on either type of ETT. Therefore, no antibiotic resistance testing was carried out. The level of bacterial colonization on the ETT surfaces was similar for both ETT types, approximately 10^1^ to 10^3^ CFU/cm^2^, see Figs. 1 and 2. Due to a large inter-patient variation for both tubes, a reduction of bacterial adhesion of the same magnitude as the one observed *invitro* was not possible to detect. As the *Alpha-haemolytic Streptococci* and *Neisseria species* were the most commonly isolated bacteria from the tubes, they were plotted/visualized in graphs, see Figs. 1 and 2.

**Table 6:** Microbiological isolation from endotracheal tubes

Microorganism	Coated ETT,	Standard ETT,
	*n* = 19 (%)	*n* = 10 (%)
*Alpha-hemolytic Streptococcus*	18 (95)	10 (100)
*Neisseria species*	11 (58)	8 (80)
*Enterococci species*	1 (5)	0 (0)
*Haemophilus parainfluenzae*	2 (11)	0 (0)
*Coagulase-negative Staphylococci*	4 (16)	1 (10)

## Discussion

This was a single-centre, randomised, single-blinded, and controlled, first- in- man, post Conformity Euro-permit (EC) certification and CE marking study. The study was planned to address a *clinical* concern that the tracheal mucosa; known to be one of the bodies most sensitive and reactive tissue; might be irritated by the new coating. The tracheal surface is known to react with coughing, swelling, and irritations to many substances even in very tiny doses. So therefore, the primary endpoint in this study was; *tolerability of the coated tube in the human trachea*.

The major finding in this comparative clinical device-testing study of coated BIP ETTs and uncoated ETTs, was that practically no differences in tolerability and performance could be detected during short term use, i.e., intubation 3 to 8 h, in patients scheduled for elective abdominal surgery during general anaesthesia. This observational first-in-man study had a randomised, single-blind and controlled design, where standard, uncoated ETTs served as comparator. The primary objective was to determine the tolerability of the BIP ETT compared to the standard ETT. Symptoms from mouth, throat and airway were assessed by questionnaires and the tracheal mucosa was inspected post exposure. Furthermore, performance and a few safety aspects related to BIP ETT were assessed based on reported AEs and adverse device-related events, as well as problems during the post-operative course, and ETT handling problems experienced by the health care personnel.

### Tolerability and adverse events

The modified EORTC QLQ H&N35 is comprised of 8 questions pertaining to symptoms/problems from the ear, nose and throat areas. The questionnaire EORTC QLQ H&N35 was primarily developed to investigate health-related quality of life issues in patients with head and neck cancer and is very well validated and used in many studies [23, 24]. We chose eight questions as they covered symptoms that may occur after an intubation. The frequency of symptoms was as expected an increase in both groups, and some differences in cough and dry mouth was observed. The difference should be treated with caution because of small study groups in combination with a statistical model, which could not correct the differences in baseline (before surgery) due to non-convergence. No known attributes of the BIP ETT have been identified that rationally could explain the possibly increased problems with cough and dry mouth. However, the design of the two ETTs differed slightly, which could potentially have played a role.

Further, there was no statistically significant difference regarding the appearance of the tracheal mucosa between the 2 treatment groups at extubation. Safety was also evaluated by AE and ADE incidence and the occurrence of any post-operative. No severe AEs were noted post-operatively in either of the treatment groups.

### Microbial assessment

In order to assess microbial adhesion to the ETTs a distal piece of the ETT was cut off after extubation. The quantity and type of pathogen and non-pathogen bacteria were assessed. The microbiological testing found *Enterococci*, *Haemophilus parainfluenzae*, *Neisseria*, *Staphylococci*, and *Streptococci species*, which are species all recognized as normal flora in the airways [25–27]. No pathogenic species were found therefore no antibiotic resistance testing was carried out.

The level of bacterial colonization on the ETT surfaces was similar for both types of tubes, approximately 10^1^ to 10^3^ CFU/cm^2^ and overall low compared to other studies where the tracheal colonization has been investigated [25–27]. We found a large inter-patient variation with regards to microbial adhesion for both types of surfaces and it is known that the normal flora differs between patients with regards to both the species found and the amount of each species. *In vitro* experiments have shown significant reduction in adhesion of several species, both Gram positive and Gram negative to the coated BIP ETT, (unpublished data).

The time of intubation in the current study was between 3 and 8 h, (mean 5 h and 14 min) which is short when evaluating microbial adherence *in vivo*. Bacterial biofilms have been shown to develop on ETTs after around 24 h, and it is this advanced form of microbial adhesion and subsequent detachment that play a part in the development of infection [28]. Further, the definition of VAP (Ventilator Associated Pneumonia) requires a time frame of at least 24 of intubation [29]. In conclusion, the used microbial assessments were found easy to employ, and detected reasonable bacterial colonisation even after short periods of intubation.

### Clinical relevance/importance, study limitations and future studies

The primary purpose of this study was to establish whether the noble metal alloy coated BIP ETT could be well tolerated during short-term (up to 8 h) clinical use in anaesthesia and elective surgery [30, 31]. This was considered necessary before further trials of the device could be carried out in a potentially more complex study group consisting of ICU patients, with longer intubation periods. Knowing the degree of sensitivity in the trachea a patient centred outcome seemed reasonable. As the human trachea had not been exposed to the coating before there was no observation that could be used as a base for a proper power analysis. We considered an observational study on at least 16 patients with 8 controls to be reasonable. As we were eager not to have fewer observations than that, we screened and included 30 patients expecting a drop-out rate of 20 %. However we ended up with a drop-out rate of only 3 %. To add an explanatory measure to eventual patient-experienced symptoms we performed a pre- and post-intubation bronchoscopy.

A methodological limitation in the study is that we intubated the patients after bronchoscopy, using the previously inserted bronchoscope to guide the tube into the trachea. This could lead to contamination of the tube and the trachea with bacteria from the mouth. Despite the possibility of contamination by the bronchoscope, the identified bacteria do not appear of clinical relevance. We do not think it has affected our results as the study was randomized, there was a control group and the same methodology was used in both groups. Other limitations of the current study include the small sample size, uneven treatment group sizes and the short intubation time and unblended inspection of the tracheal mucosa. Hence, the next step is to carry out a study in a larger population, with a longer intubation period (>24 h) and in a patient group during ICU care to enable evaluation of a possible reduction in VAP.

## Conclusions

We compared tolerability and functional performance between a new, coated endotracheal tube (BIP ETT) and a standard endotracheal tube (ETT), and found minor differences during short-term use, 3 to 8 h of intubation. The differences were observed only in 2 of 8 symptoms - in coughing, *p* = 0.022 and possibly in dry mouth (*p* = 0.014, but with presence of statistical confounders), while there was no difference in adverse events and no mucosal damage. The results should be treated with caution due to a small study size. Further, the current study had too short intubation periods and too small sample sizes to detect differences in the reduction of bacterial adhesion and biofilm formation due to large inter-individual variability in the results. In summary, the study shows that the BIP ETT is well tolerated and has good performance. Studies of larger sample sizes and longer intubation periods (>24 h) in the ICU-setting can now be planned for the evaluation of the possible efficacy of the BIP ETT in reducing bacterial colonisation and prevention of VAP.

### Key messages

Healthcare associated infections continue to play a major role in complicating hospital care. The surface of various devices can be modified in order to reduce bacterial colonization and subsequent infection. We compared new surface-coated endotracheal tubes to standard devices during elective surgery for 3–8 h, and found insignificant differences in tolerability and performance. Only larger studies with longer exposure times in special patient populations (ICU) could demonstrate possible benefits of the surface coating in reduction of VAP and produce experience supporting extrapolation of conclusion on safety from this limited study into general patient population.

## Abbreviations

AE: Adverse Events
BIP: Bactiguard Infection Protection
CE: Conformité Européenne
CFU: Colony Forming Units
EORTC: European Organization for Research and Treatment of Cancer
ETT: Endotracheal Tube
GCP: Good Clinical Practice
HAI: Hospital Acquired Infections
ICU: Intensive Care Units
NSAID: Non-Steroid Anti-Inflammatory Drugs
PBS: Phosphate Buffered Saline
PCG: Pharma Consulting Group
QLQ-H&N35: Quality of Life Head and Neck Module
SAE: Serious Adverse Events
SADE: Serious Adverse Device related Events
VAP: Ventilator Associated Pneumonia
